# New Approaches to Overcome Transport Related Drug Resistance in Trypanosomatid Parasites

**DOI:** 10.3389/fphar.2016.00351

**Published:** 2016-09-28

**Authors:** Jose A. Garcia-Salcedo, Juan D. Unciti-Broceta, Javier Valverde-Pozo, Miguel Soriano

**Affiliations:** ^1^Unidad de Enfermedades Infecciosas y Microbiología, Instituto de Investigación Biosanitaria, ibs.GRANADA, Hospitales Universitarios de Granada – Universidad de Granada, GranadaSpain; ^2^Centro de Genómica e Investigación Oncológica – Pfizer/Universidad de Granada/Junta de Andalucía, GranadaSpain; ^3^Departamento de Agronomía, Universidad de Almería, AlmeríaSpain

**Keywords:** trypanosomatid parasites, drug transport, surface transporter, efflux pumps, drug resistance, nanocarriers

## Abstract

*Leishmania* and *Trypanosoma* are members of the Trypanosomatidae family that cause severe human infections such as leishmaniasis, Chagas disease, and sleeping sickness affecting millions of people worldwide. Despite efforts to eradicate them, migrations are expanding these infections to developing countries. There are no vaccines available and current treatments depend only on chemotherapy. Drug resistance is a major obstacle for the treatment of these diseases given that existing drugs are old and limited, with some having severe side effects. Most resistance mechanisms developed by these parasites are related with a decreased uptake or increased efflux of the drug due to mutations or altered expression of membrane transporters. Different new approaches have been elaborated that can overcome these mechanisms of resistance including the use of inhibitors of efflux pumps and drug carriers for both active and passive targeting. Here we review new formulations that have been successfully applied to circumvent resistance related to drug transporters, opening alternative ways to solve drug resistance in protozoan parasitic diseases.

## Introduction

Parasitic protozoa are responsible for some of the most prevalent diseases in humans, threatening the lives of nearly 1.250 million people around the world (Lozano et al., 2012). Among them, trypanosomatids are responsible for diseases such as human African trypanosomiasis, Chagas disease and leishmaniasis (**Table 1**). Together, these neglected diseases cause more than 52.000 fatalities annually and the loss of 5 million disability-adjusted life years in developing countries worldwide (Lozano et al., 2012; Hotez et al., 2014).

**Table 1:** Trypanosomatid diseases.

Disease	human African trypanosomiasis	Visceral leishmaniasis	Chagas disease
Causative agents	*Trypanosoma brucei gambiense* *Trypanosoma brucei rhodesiense*	*Leishmania donovani* *Leishmania infantum*	*Trypanosoma cruzi*
Areas of endemicity	West and central Africa (*T. b. gambiense*) East and southern Africa (*T. b. rhodesiense*)	India, Bangladesh, Nepal, Sudan, Ethiopia, and Brazil	Central and South America
Deaths per annum	~10 000	~30 000	~12 000
Current front-line therapies	Early stage: pentamidine and suramin Late stage: melarsoprol, eflornithine monotherapy a and NECT (nifurtimox-eflornithine combination therapy)	Amphotericin B and lipid formulations Miltefosine Pentavalent antimonials Paromomycin	Acute: nifurtimox and benznidazole Indeterminate and chronic stages: no standard treatments
Transporters associated to resistance	Aquaglyceroporin 2 (pentamidine and melarsoprol) TbAAT6 (eflornithine) ABC transporters (nifurtimox)	ABC transporters: PRP1 (pentamidine and antimonials) Aquaglyceroporin 1 (antimonials) LdMT (miltefosine)	ABC efflux pumps

Despite their high prevalence, there is no effective vaccine against any of these pathogens and their treatment is based only on chemotherapy. However, the actual number of effective drugs is very low, most of them having high toxicity, a situation that has worsened by the development of resistance to almost all of them. Even the newest drugs are being compromised by the emergence of resistance in parasitic trypanosomatids (Cojean et al., 2012; Zingales et al., 2015).

The recent call for elimination and eradication of neglected tropical diseases requires research from multiple fronts, including developing strategies for the efficient delivery of current medicines and to overcome drug resistance.

## The Diseases and Their Pathogenesis

### Human African Trypanosomiasis

African trypanosomiasis is a vector-borne disease that threatens millions of people living in impoverished rural parts of sub-Saharan Africa, where they are at risk of infection. It is caused by the flagellated protozoa *Trypanosoma brucei* and transmitted by tsetse flies of the genus *Glossina*. The subspecies *T. b. gambiense* and *T. b. rhodesiense* cause human African trypanosomiasis, also called sleeping sickness (**Table 1**). In addition, the parasite infects domestic animals, causing nagana, a devastating disease of livestock in Africa. Probably, no other disease except malaria, HIV and tuberculosis, has hindered the development of a continent as has trypanosomiasis in Africa. The disease presents two stages, the early stage or hemolymphatic phase and the late stage or neurological phase which is characterized by invasion of the central nervous system. The East African variant is a chronic disease which takes years to progress, while West African trypanosomiasis is an acute infection in which the late stage develops in a few weeks or months after infection (Welburn et al., 2016). Human African trypanosomiasis is fatal if there is no a chemotherapy intervention (Matthews, 2015).

### Leishmaniasis

Leishmaniasis is a vector-borne disease caused by different species of the genus *Leishmania*, (trypanosomatidae family) and transmitted by phlebotomine sandflies. Clinical manifestations vary from local skin lesions and destructive mucosal inflammation to disseminated visceral infection (kala-azar), depending on infecting species and host factors (Murray et al., 2005; Tripathi et al., 2007). Epidemiology and immunopathology are also diverse with multiple endemic regions in areas of the tropics, subtropics, and southern Europe. A total of 350 million people are considered at risk with an estimated 1.5–2 million new cases per year, up to 500.000 of which are visceral and about 1.5 million are mucocutaneous. Visceral leishmaniasis is fatal if untreated, causing 20.000 to 30.000 deaths annually (**Table 1**), whereas cutaneous leishmaniasis has a tendency to spontaneously self-cure (den Boer et al., 2011). All forms of the disease share three pathogenetic features: tissue macrophages are targeted cells and support intracellular parasite replication; the host immunoinflammatory response regulates the outcome of disease; and there is a persistent tissue infection (Murray et al., 2005). Due to its higher impact on global health we will focus in this review on visceral leishmaniasis and the *Leishmania* species responsible for the disease (*L. donovani* and *L. infantum*).

### Chagas Disease

One of the most serious health problems in the American continent is Chagas disease, caused by the intracellular parasite *T. cruzi* (**Table 1**). Infection is transmitted by reduviid insect vectors, but can also result from vertical transmission from mother to fetus (Carlier et al., 2015), by oral ingestion of contaminated food or drink (Noya et al., 2015), blood transfusions (Young et al., 2007) and organ transplants (Kun et al., 2009).

Chagas disease is endemic to Latin America where more than 6 million people are infected, and as a result of migration is an emerging disease in traditionally non-endemic countries (Coura and Vinas, 2010; Gascon et al., 2010). While infection can remain asymptomatic for many years, an estimated 30% of individuals infected with *T. cruzi* develop potentially fatal cardiomyopathy, and gastrointestinal tract lesions (Nunes et al., 2013).

## Current Treatments

Human African trypanosomiasis therapy relies only on four drugs: pentamidine, suramin, melarsoprol and eflornithine, also known as DFMO and treatment is dependent on the subspecies and disease stage (**Table 1**) (Delespaux and de Koning, 2007; Babokhov et al., 2013). Pentamidine and suramin are used to treat early stage trypanosomiasis, when the parasite is restricted to the blood/lymphatic system. Pentamidine is the first-line treatment for *T. b. gambiense* infections and suramin covers the treatment of *T. b. rhodesiense* trypanosomiasis. Neither of these compounds crosses the blood-brain barrier, being useless to treat central nervous system infections.

Melarsoprol and eflornithine are used in the late stage of the disease, once the parasite has invaded the central nervous system. Melarsoprol was introduced in the mid-20th century and is currently the only effective drug against the late stage of human African trypanosomiasis caused by both subspecies. Eflornithine was the last drug to be introduced to treat human African trypanosomiasis 50 years ago. The drug crosses the blood-brain barrier but lacks effectiveness against *T. b. rhodesiense*.

The recently launched eflornithine-nifurtimox combined therapy is the safest treatment for late-stage trypanosomiasis (Priotto et al., 2009; Babokhov et al., 2013). The combination of eflornithine and nifurtimox allows significant reduction of eflornithine dose and treatment duration. However, like eflornithine alone, this combined therapy is only effective in the second stage of *T. b. gambiense* infections. This leaves melarsoprol, an arsenical derivate that causes reactive encephalopathy in about 10% of treated patients, as the only effective drug against both *T. b. rhodesiense* and *T. b. gambiense* late-stage infections. Because of this, the increasing rate of melarsoprol treatment failures is alarming.

In the case of visceral leishmaniasis, the commercially available first-line drugs for the treatment of the disease are: pentavalent antimonials, amphotericin B, miltefosine, pentamidine and paromomycin (**Table 1**) (Monge-Maillo and Lopez-Velez, 2013; Elmahallawy and Agil, 2015). Like human African trypanosomiasis, visceral leishmaniasis treatment regimens are based on *Leishmania* species and geographic region.

There are only two drugs now available for Chagas disease, nifurtimox and benznidazole (**Table 1**). Both are very effective if given soon after infection. However, treatment failures are not infrequent (Munoz et al., 2013; Pinto et al., 2013) and both drugs cause adverse effects (Jackson et al., 2010). Benznidazole is the first choice for Chagas disease treatment due to lower side effects than nifurtimox.

## Mode of Action, Surface Transporters and Resistances

**FIGURE 1:**
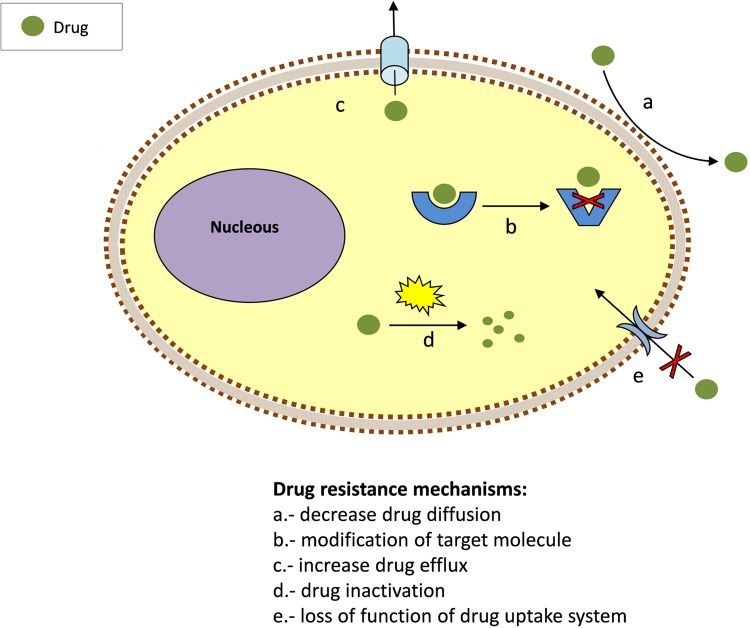
**Common mechanisms for drug resistance**.

To exert its action, a drug must be able to interact inside the cell with its target/s at a concentration sufficient to inhibit its biological activity. The emergence of resistance is associated with this process. Parasites have evolved numerous ways to overcome the toxicity of drugs. Most of them are based on reducing the drug concentration that can access the target or, alternatively, modifying the target molecule. Thus, drug resistance might involve mutations causing the loss of their uptake system (**Figure 1**). Once inside, drugs may be modified and inactivated, excreted or relocated into vacuoles. Prodrugs require activation to become effective and suppression or loss of the activating process may lead to resistance. Another mechanism of drug resistance is the alteration of drug/target interaction by increasing the number target molecules or their modification. Trypanosomatids employ these strategies to develop resistance.

**FIGURE 2:**
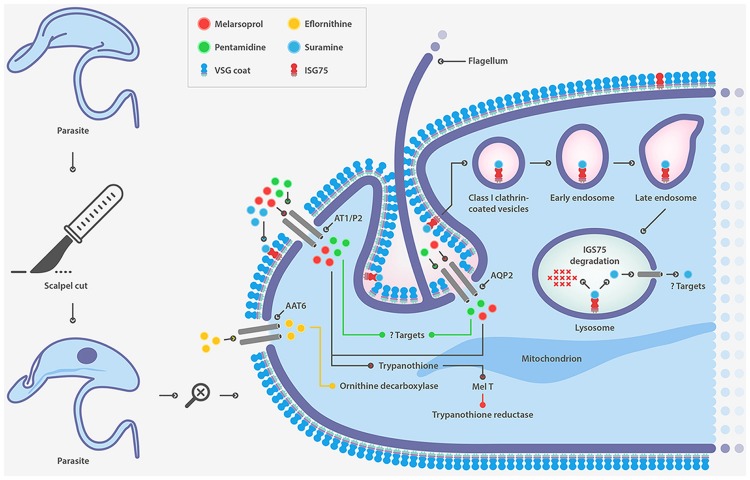
**Drug transporters in *T. brucei***.

**Suramin**, a polysulphonated naphthylamine-based compound, is a highly negatively charged molecule that accumulates inside the trypanosome probably bound to human serum proteins such as lipoproteins, albumin, globulins and fibrinogen (Vansterkenburg et al., 1993). The mode of action of this drug is not well established and is attributed to inhibition caused by electrostatic interactions with essential proteins, enzymes etc. The manner in which the drug enters the cell was postulated two decades ago to be receptor-mediated endocytosis, but it was only recently that evidence in this regard has been provided. Following an RNAi approach, several genes whose down-regulation confers resistance to suramin have been identified (Alsford et al., 2012). Surprisingly, most of these genes code for proteins that are part of the endocytic machinery and the endosomal compartment. This finding has led ISG75 being proposed as the major surface receptor for suramin uptake (**Figure 2**). Although suramin was the first drug used to treat trypanosomiasis early last century, resistance cases in the field have not yet been reported.

**Pentamidine** is a diamine that rapidly accumulates inside trypanosomes against a concentration gradient up to millimolar concentrations (Damper and Patton, 1976a,b; Berger et al., 1995). It is known that pentamidine binds DNA, accumulates in the mitochondria and collapses the mitochondrial membrane potential but its precise mode of action has not been yet satisfactory resolved.

**Melarsoprol**, an arsenical derivate, is unstable once in circulation in the human body and is rapidly processed to the more stable form melarsen oxide (Keiser et al., 2000). Once inside the parasite, melarsen oxide reacts with trypanothione, a metabolite exclusive to trypanosomes which is responsible for detoxification of peroxides and for the redox balance of the cell forming a toxic adduct known as MeltT.

It is well established that pentamidine and melarsoprol enter the cell using the same transporter. The adenosine/adenine transporter P2 was the first described transporter associated with melarsoprol (**Figure 2**) (Carter and Fairlamb, 1993; Maser et al., 1999) and pentamidine uptake (Carter et al., 1995; De Koning, 2001). However, deletion of the gene generated a cell with marginal resistance to both drugs (Matovu et al., 2003). Recently aquaglyceroporin 2, a member of a family of surface channel proteins involved in the transport of water and small non-charged solutes, has been identified as the transporter responsible for resistance to high concentrations of pentamidine and melarsoprol (Alsford et al., 2012; Baker et al., 2012).

Although resistance to pentamidine has not been officially reported, melarsoprol resistance is a common issue. A recent study on *T. b. gambiense* demonstrated that rearrangements of the *aquaglyceroporin 2/3* locus accompanied by *aquaglyceroporin 2* gene loss also occurs in the field, and that *T. b. gambiense* carrying such mutations correlates with a resistance to pentamidine and melarsoprol (Graf et al., 2013). Pentamidine failures have commonly been dismissed as misdiagnosis of late-stage sleeping sickness. Thus, we speculate that resistance to pentamidine is likely present in the field.

Pentamidine has also been used as an alternative therapy for antimonial-refractory patients in visceral leishmaniasis. The antileishmanial mechanisms of pentamidine include inhibition of polyamine biosynthesis by inhibiting arginine and polyamine uptake (Basselin et al., 2000), DNA minor groove binding (Basselin et al., 1998), and inhibition of respiratory chain complex II (Mehta and Shaha, 2004). Thus, the main target for the drug seems to be the parasitic mitochondria.

**FIGURE 3:**
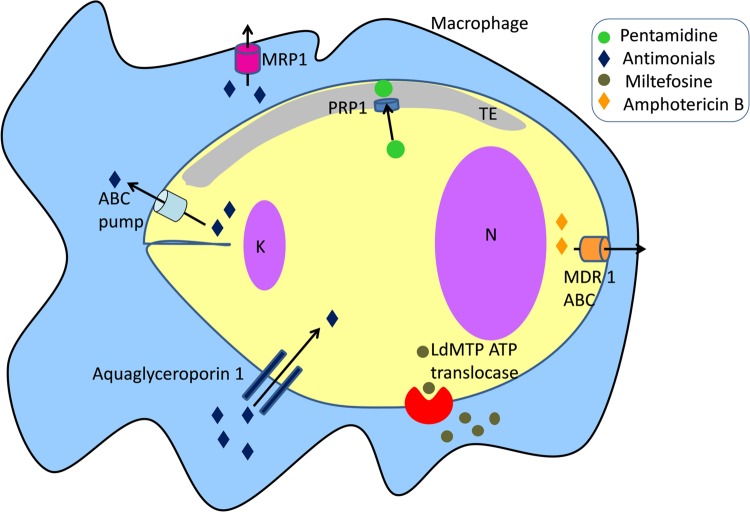
**Drug transporters in *Leishmania.* K, kinetoplast; N, nucleus**.

Resistance to pentamidine in *Leishmania* has been reported but the mechanisms are not clearly defined. Pentamidine resistance protein 1 (PRP1), a member of the ATP-binding cassette (ABC) transporter superfamily plays a role in resistance to pentamidine and cross-resistance to trivalent antimonials in *Leishmania* when over expressed (**Figure 3**) (Coelho et al., 2003, 2006, 2007, 2008). PRP1 is an intracellular protein possibly associated with the tubulovesicular element that is linked to the exo- and endo-cytosis pathway (McConville et al., 2002; Coelho et al., 2006).

**Eflornithine**, is an inhibitor of ornithine decarboxylase, an essential enzyme for the first step of polyamine synthesis and the formation of trypanothione (Bacchi et al., 1980). The drug crosses the blood-brain barrier and its entry into trypanosomes is also transporter-mediated. Several groups have recently identified the amino acid transporter TbAAT6 as responsible for eflornithine uptake showing that its lost is associated to drug resistance (**Figure 2**) (Vincent et al., 2010; Baker et al., 2011; Schumann Burkard et al., 2011). However, this mechanism of resistance to eflornithine still remains to be confirmed in resistant parasites from clinical isolates.

**Pentavalent antimonials** are considered as pro-drugs which are further converted to trivalent antimonials or antimonites, the active forms of the drug. The mechanism of action seems to be multifactorial, including inhibition of macromolecular biosynthesis (Berman et al., 1985), likely by targeting glycolysis and fatty acid oxidation (Berman et al., 1987). DNA fragmentation and externalization of phosphatidylserine on the outer surface of the plasma membrane have been also reported (Sereno et al., 2001; Lee et al., 2002; Sudhandiran and Shaha, 2003). However, the specific targets in these pathways have not been identified. Metabolism of thiol is essential in the mechanism of action of antimonials. Trypanothione is the major thiol in *Leishmania*. Antimonites exhibit *in vitro* inhibition of trypanothione reductase, an enzyme responsible for protection of parasites from host reactive oxygen and nitrogen species (Wyllie et al., 2004).

The route of entrance of antimonial drugs into *Leishmania* remains elusive. The surface transporter aquaglyceroporin 1 has been shown to facilitate trivalent antimonial entrance, and its overexpression renders *Leishmania* cells hypersensitive to antimonials (Gourbal et al., 2004).

Mechanisms responsible for antimonial acquired resistance in clinical isolates have been studied for several decades, but the results are puzzling. Clinical isolates are characterized by presenting high variability in the mechanism as well as in the degree of resistance (Mukherjee et al., 2007). Resistant *Leishmania* cells to antimonials have been selected *in vitro* and some resistance mechanisms have been suggested, including reduced accumulation (Dey et al., 1994) and loss of reduction of the pentavalent metal (Shaked-Mishan et al., 2001).

ABC transporters use the hydrolysis of ATP to translocate a variety of compounds across biological membranes. Some ABC transporters play a major role in resistance of tumors to anticancer drugs (Fletcher et al., 2016), and antibiotic resistance in pathogenic microorganisms (Klein et al., 2011; Leprohon et al., 2011). Previous studies have reported the role of ABC transporters in *Leishmania*-acquired antimonial resistance (**Figure 3**). Gene amplification of ABC pumps has been associated with resistance to antimonials in laboratory-derived and clinical isolate-resistant parasites (Ferreira-Pinto et al., 1996; Haimeur and Ouellette, 1998; Haimeur et al., 2000; Mukherjee et al., 2007; Mandal et al., 2009; Manzano et al., 2013, 2014; Perea et al., 2016). Overexpression of ABC transporters regulates the elimination of antimonites through two different routes: their sequestration into the intracellular vacuolar compartment or direct efflux across the plasma membrane. A different mechanism includes the modulation of macrophage drug transporters and metabolizing enzymes related to resistance to antimonials. Infection with an antimonite-resistant *Leishmania* strain upregulated multidrug resistance-associated protein 1 (MRP1) and permeability glycoprotein (P-glycoprotein) in host cells, leading to the inhibition of intracellular drug accumulation by reducing antimonite influx (**Figure 3**) (Mukherjee et al., 2007; Mandal et al., 2009). Antimonial resistance is also associated with aquaglyceroporin 1 transporter. Down-regulation of aquaglyceroporin 1 is correlated with lower antimonite uptake, leading to a decrease in drug concentration within the parasite (**Figure 3**) (Marquis et al., 2005).

**Amphotericin B** is a polyene drug with selective activity against fungi, *Leishmania* and *T. cruzi* due to its higher affinity for ergosterol, the predominant sterol in these pathogens over cholesterol, the predominant sterol in mammalian host cells. Amphotericin B induces the formation of small membranous pores that alter membrane permeability toward cations, water, and glucose molecules (Ramos et al., 1996).

Mechanisms of drug resistance to amphotericin B developed by *Leishmania* are various and include: (i) a change in membrane fluidity; (ii) a decrease in thiol and reactive oxygen species levels likely due to a hyperactivity tryparedoxin cascade; and (iii) an increase in drug efflux from the cell (Purkait et al., 2012). An RT-PCR analysis of clinical isolates of *L. donovani* has shown the upregulation of several genes implicated in trypanothione biosynthesis and the tryparedoxin cascade. Furthermore, the mRNA level of the ABC transporter *MDR1* was found to be four-fold higher in R-strains which supported the observations for increased drug efflux (**Figure 3**) (Purkait et al., 2012).

**Miltefosine** (hexadecylphosphocholine) is a membrane-active alkyl phospholipid that was originally developed as an anticancer drug. Currently, it is used as the first-line treatment for visceral leishmaniasis and was considered a major step forward in antileishmanial therapy (Jha et al., 1999; Sundar et al., 2006). The precise mode of action remains largely unknown. Miltefosine reduces the lipid content and augments the phosphatidylethanolamine content in the parasite membrane, suggesting a partial inhibition of phosphatidylethanolamine-N-methyltransferase which causes a delay in cell proliferation (Loiseau and Bories, 2006). Miltefosine also triggers an apoptosis-like cell death in *Leishmania* (Paris et al., 2004; Alzate et al., 2008; Khademvatan et al., 2011; Marinho Fde et al., 2011) but it is unknown how this happens.

Miltefosine activity is due to intracellular accumulation of the drug, which is regulated by two transporters, the miltefosine transporter LdMT, a novel P-type ATPase member of the aminophospholipid translocase family and its beta subunit LdRos3 (**Figure 3**) (Perez-Victoria et al., 2003; Perez-Victoria F.J. et al., 2006). Reported resistances are associated with a severely diminished intracellular drug concentration, due to the overexpression of efflux pumps and, mainly, to the failure of miltefosine transporters (Perez-Victoria J.M. et al., 2006). Furthermore, low expression of the *LdRos3* subunit is the cause of natural resistance to miltefosine observed in *Leishmania* (Sanchez-Canete et al., 2009). Clinical resistances have been recently isolated and most of them are associated with punctual mutations in the LdMT transporter (Cojean et al., 2012).

**Paromomycin** (aminosidine) is an aminoglycoside produced by *Streptomyces rimosus* with both antibacterial and antileishmanial activity (No, 2016). The drug was licensed in 2006 and field resistance has not yet been reported.

**Nifurtimox** and **benznidazole** are nitroheterocyclic drugs carrying a nitro group linked to an aromatic ring that function as prodrugs and must undergo a reductive activation within the parasite to have cytotoxic effects. This reaction is catalyzed by NADH-dependent bacterial-like nitroreductase and drug-induced resistant parasites arise as a consequence of mutations in this enzyme (Wilkinson et al., 2008; Campos et al., 2014). In addition, P-glycoprotein efflux and P-glycoprotein ATPase activity have been described in *T. cruzi* resistant to nitroheterocycles, implicating ABC efflux pumps in drug resistance (Campos et al., 2013). Resistance to nitroheterocycles seems to be due to qualitative differences in P-glycoprotein function (Murta et al., 2001) and *P-glycoprotein* gene overexpression (Franco et al., 2015; Zingales et al., 2015).

## Strategies to Specifically Overcome Transport Related Drug Resistance

### Efflux Pumps Inhibition

Among the different drug resistance mechanisms previously described, those based on decreasing the drug concentration at the target site by drug movement through the membranes appear to play an important role in *Leishmania* and *T. cruzi*. Hence, inhibition of the activity of ABC transporters represents an interesting strategy for controlling drug resistance.

A number of compounds have been identified to inhibit P-glycoproteins which are as diverse as calcium channel blockers, calmodulin antagonists, protein kinase inhibitors, antibiotics, hydrophobic peptides, hormone derivatives, and flavonoids. Their effectiveness in reversing acquired drug resistance in *Leishmania* varies greatly between species and cell cycle stages due to marked differences in the expression of their target, ABC transporters.

Verapamil, a calcium channel blocker, has been employed to reverse drug resistance to antimony associated with the overexpression of several P-glycoproteins and MRP in *Leishmania* and *T. cruzi* strains obtained in the laboratory (Neal et al., 1989; BoseDasgupta et al., 2008) and from clinical isolates of *Leishmania* (Valiathan et al., 2006). Verapamil is also able to overcome resistance to pentamidine in *Leishmania* (Basselin et al., 2002; Coelho et al., 2008) but its effectiveness is species dependent (Mukherjee et al., 2006).

Calmodulin antagonists have been shown to reverse drugs resistance in parasites. Phenothiazine derivates such as chlorpromazine increased sensitivity to antimonials in multidrug resistant *L. donovani* and *L. major* strains *in vitro* (el-On et al., 1986). These compounds have antiprotozoal activity *per se* (Pearson et al., 1984; Werbovetz et al., 1992). However, the study of phenothiazine derivates has been abandoned due to the high rate of toxicity founded in its leading molecule chlorpromazine.

Statins, also known as HMG-CoA reductase inhibitors, are drugs currently used to lower the level of low-density lipoprotein (LDL) in the blood preventing atherosclerosis and cardiovascular disease. Besides this lipid-lowering activity, statins also inhibit ABC transporters. Lovastatin, a member of the statin family, in combined therapy with the antifungal drug miconazole, reduced *Leishmania* cell growth and macrophage infection, although the proposed mechanism of action by lovastatin was through inhibition of sterol biosynthesis (Haughan et al., 1992). Lovastatin has been shown to inhibit MRP1 and P-glycoprotein in *L. donovani* allowing antimony accumulation and parasite killing within macrophages (Mookerjee Basu et al., 2008). Moreover, pretreatment with lovastatin in hamsters infected with an antimonial-resistant *L. donovani* cell line reversed acquired resistance to antimonials.

Flavonoids constitute a class of natural inhibitors of P-glycoprotein and related ABC transporters in *Leishmania* (Perez-Victoria et al., 1999; Wong et al., 2007; Kennedy et al., 2011). Quercetin, a flavonoid that regulates human ABC transport by reducing P-glycoprotein synthesis reduced miltefosine accumulation in *Leishmania* (Castanys-Munoz et al., 2007). Synthetic flavonoid dimmers have been used to reverse drug resistance to antimony and pentamidine in *L. donovani* by increasing intracellular drug accumulation (Wong et al., 2007).

Sesquiterpenes are natural products that overcome multidrug resistance in *Leishmania* including resistance to miltefosine. Sesquiterpene C3 fully sensitized a multidrug resistant *Leishmania* by increasing intracellular drug concentration through the modulation of new ABC transporter activity (Perez-Victoria et al., 2001).

### Drug Transporters

**FIGURE 4:**
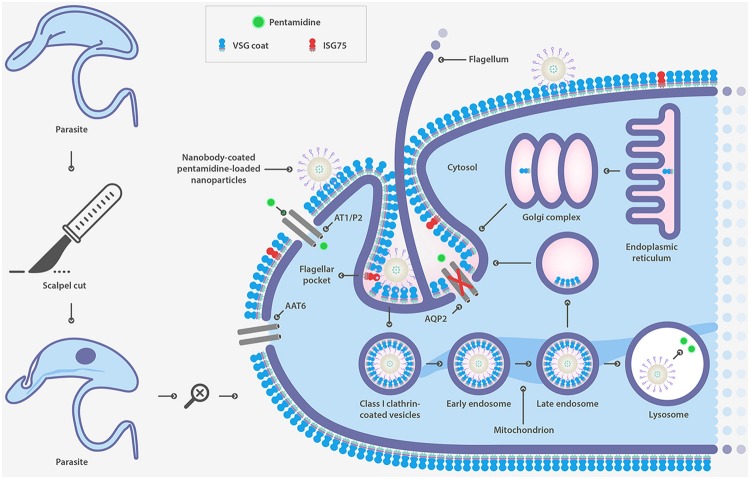
**Strategy to overcome drug resistance associated to mutations in surface transporter in *T. brucei* based on nanobody-coated pentamidine loaded nanoparticle**.

A different strategy to defeat drug resistance associated with surface transporters is the use of drug delivery systems. Nanoparticles of biodegradable and biocompatible polymers are widely used for this purpose. Specific cell-surface targeting has been shown to be an effective strategy to overcome drug resistance associated with the loss of function of surface transporters responsible for drug uptake in *T. brucei*. PEGylated PLGA nanoparticles were coupled to a single-domain heavy-chain antibody fragment (nanobody) that specifically recognizes the surface of the parasite (**Figure 4**) (Stijlemans et al., 2004). Nanoparticles were loaded with pentamidine, the first-line drug for *T. b. gambiense* acute infection. An *in vitro* and *in vivo* effectiveness assay demonstrated that the formulation was able to significantly reduce the effective drug concentration (Arias et al., 2015). Moreover, an improved version of this nanocarrier reduced the curative dose 100-fold and most significantly, circumvented drug resistance as a result of mutations in aquaglyceroporin 2 (Unciti-Broceta et al., 2015), the surface channel protein that mediates pentamidine uptake in *T. brucei* (Baker et al., 2012). This study represents a proof-of-concept as a strategy for overcoming drug resistance linked to the loss of function of surface transporters.

Cell-penetrating peptides, also known as peptide transduction domains, membrane translocating sequences and Trojan peptides, are short peptides with the ability to enter almost any cell. In the late 1980s it was observed that the trans-activator of transcription (TAT) of the HIV virus as well as a chemically synthesized version of TAT was able to enter cells (Frankel and Pabo, 1988; Green and Loewenstein, 1988). Cell-penetrating peptides are highly cationic and usually rich in arginine and lysine amino acids. Their exceptional property of delivering into the cell a wide variety of cargoes such as proteins, oligonucleotides and even 200 nm liposomes has made them attractive candidates to transport drugs to the interior of cells (van den Berg and Dowdy, 2011). The cargo is associated with the peptides either through chemical linkage via covalent bonds or through non-covalent interactions, and they get inside cells primarily via endocytosis but also by direct membrane penetration.

Conjugation of miltefosine to the cell penetrating peptide TAT has been applied as an efficient strategy to defeat drug resistance associated with lack of function of the LdMT surface transporter in *Leishmania* (Luque-Ortega et al., 2012). Functionalized miltefosine carrying a thiol group was conjugated to TAT_48-60_ through either a disulphide or a thioether bond. Drug conjugates were able to enter and kill both promastigote and intracellular amastigote forms of a *Leishmania*-resistant strain. Intracellular release of the drug was not a requirement since the most metabolically stable thioether conjugate retained significant leishmanicidal activity compared to the less stable disulphide conjugate.

The strategy was also applied to kill *T. brucei*, a parasite naturally insensitive to miltefosine due to the lack of the surface translocase system responsible for drug uptake (Luque-Ortega et al., 2012). This report provides proof-of-mechanism for the use of cell-penetrating peptide conjugates to bypass drug resistance due to diminished intracellular drug concentration in parasites, and open the possibility to extend chemotherapy into other parasites intrinsically devoid of membrane translocation systems.

## Strategies With Potential to Overcome Resistance

Cyclodextrins are cyclic oligosaccharides, with a hydrophilic outer surface and a hydrophobic interior cavity. Cyclodextrins are able to form water-soluble inclusion complexes with many poorly soluble lipophilic compounds, and have been widely used to enhance drug solubility and reduce drug toxicity (Zhang and Ma, 2013). Melarsoprol, a highly toxic drug for treating the late stage of human African trypanosomiasis, has been successfully occluded within two different types of cyclodextrin: β-cyclodextrin and randomly methylated-β-cyclodextrin (Rodgers et al., 2011). These compounds retained trypanocidal activities *in vitro* and cured the late stage of the disease in a murine model.

The entrance route of melarsoprol-cyclodextrin complexes is probably different than that of free melarsoprol. Entry of the drug with cyclodextrin is likely via endocytosis whereas that of free melarsoprol is via the aquaglyceroporin 2 channel. As mentioned before, resistance to melarsoprol in clinical isolates correlates with mutations in aquaglyceroporin 2 surface transporter. Therefore, although it has not been tested, it is quite possible that the melarsoprol-cyclodextrin formulation could circumvent melarsoprol resistance.

Biopharmaceutical issues are more complex in the case of intracellular parasites such as *Leishmania* or *T. cruzi*. The design of drug delivery systems must take into account aspects like passage through physical barriers and cell membranes, the mechanism of cellular uptake, stability, activity and kinetics of the drug in the target cell environment. The pharmacodynamic response of antiparasitic drugs is governed in intracellular parasites by the drug concentration reached inside host cells rather than that in the circulatory system (Date et al., 2007). In general, drug delivery systems increases efficacy and reduce side effects relative to free drug. It has also been reported that these systems can increase susceptibility to drug in resistant parasites by increasing drug concentration inside targeted host cells, but there are very few specific studies about circumventing drug resistance (Yasinzai et al., 2013). However, It has been described the use of nanodevices to encapsulate alternative drugs for treatment of resistance to a drug. (Yasinzai et al., 2013; Gutierrez et al., 2016).

*Leishmania* is a good model to be targeted by these nanoformulations because it only parasitizes macrophages which are responsible for the clearance of blood particulate materials *in vivo*. Liposomes, niosomes, nanoemulsions, nanodiscs, transfersomes, solid lipid nanoparticles, polymeric and metalic micro/nanoparticles and other delivery systems have been extensively investigated for the treatment of leishmaniasis (Date et al., 2007; Gupta et al., 2010; Yasinzai et al., 2013; Gutierrez et al., 2016). Liposomes are the most frequently used drug delivery systems for leishmaniasis treatment. It is described that liposomes deliver their contents into the macrophage cytoplasm by fusing their membrane to the plasma membrane of the cells, increasing the drug concentration inside it. Different types of liposomes have been developed for both passive and active targeting of macrophages. These formulations included conventional liposomes and arsonoliposomes (containing arsenolipids with covalently linked arsenic) for passive targeting and sugar-bearing liposomes (mannose, or neoglycoprotein); positively charged liposomes (containing phosphatidyl choline or stearyl amine); peptide-grafted liposomes (with macrophage-activating peptides like tuftsin or the chemotactic peptide f-Met-Leu-Phe) and immunoliposomes (IgG-coupled liposomes) for active targeting of macrophages. A commercial preparation of Amphotericin B in liposomes (AmBisome) was approved for treatment of visceral leishmaniasis as the first choice for the treatment of resistance associated with antimonials (Ng et al., 2003). It has also been shown that liposomal miltefosine improved the susceptibility of miltefosine-resistant *Leishmania* promastigotes (Papagiannaros et al., 2005). *In vitro* investigations on arsonoliposomes revealed that they were active against amphotericin B and miltefosine-resistant *L. donovani* at relatively low concentrations of arsenolipids (Antimisiaris et al., 2003).

Various polymeric nanoparticulate or micellar systems of polyalkylcyanoacrylate, polylactic acid, poly (lactic co-glycolic acid), poly(ε-caprolactone), chitosan, albumin, gelatin, lipid and inorganic nanoparticles (gold, silver, titanium oxide, etc) were also tested for the treatment of leishmaniasis (Date et al., 2007; Gupta et al., 2010; Pham et al., 2013; Yasinzai et al., 2013; Gutierrez et al., 2016). Some particulate nanocarriers can cross biological barriers by endocitosis-like mechanisms and could be appropriate to target intracellular parasites like *Leishmania*. Ultrastructural studies indicated that nanoparticles were concentrated in the phagolysosome, quite close to *Leishmania* amastigotes, releasing the drug into the macrophage cytoplasm (Fusai et al., 1997). PLGA nanoparticles loaded with the antiparasitic drug andrographolide and stabilized the excipient vitamin E-TPGS (D-a-tocopheryl polyethylene glycol 1000 succinate) which is an inhibitor of the P-glycoprotein efflux pump showed high effectivity against wild and multidrug resistant *L. donovani* strains *in vitro* (Mondal et al., 2013). In this context, piperolactam A loaded hydroxypropyl-β-cyclodextrin nanoparticles have also proved effective against wild and multiresistant *L. donovani in vitro* (Bhattacharya et al., 2016). Previous studies showed that some metal and metal oxide nanoparticles have antimicrobial activity by generation of reactive oxygen species. These nanoparticles have been assayed against drug-resistant bacteria and *Leishmania* parasites (Allahverdiyev et al., 2011; Das et al., 2013; Jebali and Kazemi, 2013). After exposure to nanoparticles, the highest antileishmanial activity was observed for Ag-nanoparticles, followed by Au, TiO2, ZnO, and MgO nanoparticles. Both ultraviolet and infrared light increased the antileishmanial properties of all nanoparticles. Unfortunately, despite their significant potential in antimicrobial applications, the toxicity of metal oxide nanoparticles has restricted their use. Nevertheless, recent studies using crystalline nanoparticles of ZnCuO showed better biocompatibility indicating that metal oxide nanoparticles in resident non-toxic form have considerable potential for antibacterial and antiparasitic applications in the future (Nadhman et al., 2015).

Transfersome is a nanocarrier designed for drug delivery across the skin barrier. Transfersome formulations bearing amphotericin B have been tested against sensitive and resistant clinical isolates of *L. donovani* and compared with the conventional liposomal formulation and free amphotericin B. Transfersome formulations were significantly more active than conventional liposomes and free amphotericin B suggesting improved penetration and better partitioning in skin layers. Transfersomes were also able to partially reverse resistance to amphotericin B. Nevertheless, potential utilities of these novel formulations as an alternative chemotherapeutic approach for the treatment of resistant leishmaniasis necessitate further investigations (Singodia et al., 2010).

In the case of Chagas disease, there is few research works published in the context of drug delivery systems and only in some of them resistance is mentioned. There are two main challenges in Chagas disease therapy using drug carriers, the first one is to reach disseminated intracellular parasites (in cardiac, skeletal and smooth muscle, glial cells, etc.) at therapeutic doses and the second is the selectivity for target cells since current drugs are toxic. Different nano-formulations have been assayed, including liposomes, polyalkylcyanoacrylate nanospheres, poly(ethyleneglycol)-co-poly(lactic acid) nanoparticles, dendrimers, and other lipid formulations. In general, these formulations had limited efficacy relative to free drug with the only advantage of reducing toxicity (Romero and Morilla, 2010; Salomon, 2012; Morilla and Romero, 2015). Low endocytic activity of host forms of the parasite (trypomastigote and amastigote), target selectivity and the selected nanocarrier could be responsible for this lack of effectiveness. Nevertheless improvements in nanocarriers design like pH-sensitive formulations, passive and active targeting, prodrug dendrimers, nitric oxide-releasing nanoparticles, etc., could improve drug efficacy (Romero and Morilla, 2010; Morilla and Romero, 2015; Seabra et al., 2015).

## Concluding Remarks

Diseases caused by trypanosomatids are a serious health and socioeconomic problem, in particular in developing countries. The lack of effective vaccines, old and unsafe drugs, along with the emergence and spread of resistance, has recently led to the launch of campaigns supporting the battle against these diseases. However, the discovery and development of new drugs have been slowed by the high cost associated with processing new molecules through the long drug discovery pipeline together with the poor financial motivation for pharmaceutical companies. However, recent advances in the elucidation of drug resistance mechanisms have led to the development of new therapeutic approaches with the ability of restoring/improving the efficacy of the existing drugs.

In trypanosomatids, resistance mechanisms are often associated with changes in the function or quantity of surface transporters leading to a lower of drug uptake or increased efflux. The loss of function of surface transporters responsible of drug uptake is a common mechanism of drug resistance in African trypanosomes (Munday et al., 2015) and overexpression of multidrug export transporters such as efflux pumps play a key role in the emergence of resistance in *Leishmania* and to lesser extent in *T. cruzi* (Leprohon et al., 2011). Currently, both mechanisms have become the focus of research for the development of new therapeutic tools to recover previously failed compounds. The inhibition of the activity of ABC proteins has been well studied for cancer and represents an interesting way to control drug resistance associated with enhanced drug efflux in protozoan parasites. However, although drug resistance is not a serious problem for the treatment of Chagas disease, the use of efflux pumps inhibitors to reverse resistance in *T. cruzi* is scarce and deserves to be explored more deeply.

The use of an alternative drug entrance route has also proved to be a successful approach to overcome drug resistance associated with mutations that reduce drug import. Passive (cell penetrating peptides) and active (nanobodies) targeting engineered nanosystems can be used to introduce exiting drugs into the parasite that avoid the cell surface transporters involved in drug up take. Both are proofs of concept of strategies capable of reversing resistance to many first-line treatments. Efforts should be made in the design of drug delivery systems to improve the effectiveness of current nanoformulations targeting intracellular forms of *Leishmania* and *T. cruzi*.

In summary, all these novel approaches open a new way to develop and test promising anti-parasitic tools. These advances, combined with the constant evolution of the nanomedicine, may portend new effective developments in the treatment of diseases caused by trypanosomatids.

## Author Contributions

Wrote the paper: JAGS, MS. Performed the figures: JDUB, JVP.

## Conflict of Interest Statement

The authors declare that the research was conducted in the absence of any commercial or financial relationships that could be construed as a potential conflict of interest.
